# Genetics Factors in Major Depression Disease

**DOI:** 10.3389/fpsyt.2018.00334

**Published:** 2018-07-23

**Authors:** Maria Shadrina, Elena A. Bondarenko, Petr A. Slominsky

**Affiliations:** Laboratory of Molecular Genetics of Hereditary Diseases, Institute of Molecular Genetics, Russian Academy of Sciences, Moscow, Russia

**Keywords:** depressive disorder, candidate genes, brain-derived neurotrophic factor, hypothalamus-pituitary-adrenal axis, cytokines, GWAS, epigenome

## Abstract

Depressive disorders (DDs) are one of the most widespread forms of psychiatric pathology. According to the World Health Organization, about 350 million people in the world are affected by this condition. Family and twin studies have demonstrated that the contribution of genetic factors to the risk of the onset of DDs is quite large. Various methodological approaches (analysis of candidate genes, genome-wide association analysis, genome-wide sequencing) have been used, and a large number of the associations between genes and different clinical DD variants and DD subphenotypes have been published. However, in most cases, these associations have not been confirmed in replication studies, and only a small number of genes have been proven to be associated with DD development risk. To ascertain the role of genetic factors in DD pathogenesis, further investigations of the relevant conditions are required. Special consideration should be given to the polygenic characteristics noted in whole-genome studies of the heritability of the disorder without a pronounced effect of the major gene. These observations accentuate the relevance of the analysis of gene-interaction roles in DD development and progression. It is important that association studies of the inherited variants of the genome should be supported by analysis of dynamic changes during DD progression. Epigenetic changes that cause modifications of a gene's functional state without changing its coding sequence are of primary interest. However, the opportunities for studying changes in the epigenome, transcriptome, and proteome during DD are limited by the nature of the disease and the need for brain tissue analysis, which is possible only *postmortem*. Therefore, any association studies between DD pathogenesis and epigenetic factors must be supplemented through the use of different animal models of depression. A threefold approach comprising the combination of gene association studies, assessment of the epigenetic state in DD patients, and analysis of different “omic” changes in animal depression models will make it possible to evaluate the contribution of genetic, epigenetic, and environmental factors to the development of different forms of depression and to help develop ways to decrease the risk of depression and improve the treatment of DD.

## Introduction

Depressive disorders (DDs) comprise one of the most widespread forms of psychiatric pathology. According to the World Health Organization, about 350 million people are affected by a DD. The worldwide prevalence of DDs varies from 3% in Japan to 16.9% in the USA; in most countries, this prevalence ranges from 8 to 12% (1). It is predicted that, by 2020, DDs will be the second-leading cause of disability throughout the world after ischemic heart disease (2).

DD entails a number of unfavorable consequences with medical and sociological relevance, and affects significantly the quality of life and adaptive ability. Long-term and severe depression mixed with chronic somatic or neurological conditions might lead to attempted suicide.

Despite the great medical and social significance of DDs, there is no clear conceptualization to explain the causes and mechanisms of DD development. Several theories have been suggested to explain the onset of depression and have been confirmed by biochemical, immunological, and physiological studies. Parallel to well-known “monoamine,” “cytokine,” and “stress-induced” (hypothalamus–pituitary–adrenal (HPA) axis and stress theories) depression models, the phenomena of altered brain neural plasticity and neurogenesis and circadian rhythm desynchronosis (the chronobiological model) have been proposed to explain the onset of depression.

Family and twin studies have provided strong evidence for the contribution of genetic factors to the risk of depression. For instance, a meta-analysis of twin research data shows that the heritability rate for depression is 37% (95% CI: 31%−42%), and data from family studies show a two- to threefold increase in the risk of depression in first-degree offspring of patients with depression (3). Heritability has also been shown to be especially influential in severe forms of depression (4, 5). The illness severity depends on whether DDs are inherited maternally or paternally (6, 7).

Since 1978, when the first study devoted to identifying possible candidate genes related to DDs was published (8), many studies have searched for genes involved in the progression of depression worldwide. Based on the available data on the putative neurobiological mechanisms underlying DDs, more than 100 candidate genes have been analyzed to identify the possible associations between their alleles and the risk of depression onset or its symptoms. The studies of DD pathogenesis have yielded conflicting results. Development of DNA microchip technology has made it possible to conduct genome-wide associations studies (GWASs) to look for risk factors of depression onset, independent of the hypotheses to explain depression pathogenesis available at the time. However, GWASs using large sets of samples, including thousands of patients with different forms of DDs and tens of thousands of patients in meta-analyses, have failed to identify any specific loci responsible for predisposition to DDs. These studies have also not unambiguously defined the biological mechanisms underlying the pathogenesis of the pathology of DDs. This failure to identify clearly the genetic associations and underlying mechanisms indicate that depression is a complicated multifactor heterogeneous psychiatric disorder. It is likely that the predisposition to DDs is determined by the coordinated action of many genes and their interaction with each other and with diverse environmental factors. It is also likely that each gene by itself makes a relatively small contribution to the pathogenesis of the disease (9).

In this review, we discuss the principal theories to explain the development of depression and the genetic evidence in support of these theories. The final section discusses the results of GWASs and the possible contribution of epigenetic factors to the risk of onset of DDs.

## Brief characterization of depressive disorder

### Depression

Depression (lat. *depressio*—gloominess, oppression) is a psychiatric disorder characterized by a pathologically low mood (hypothymia) and negative esteem about oneself, one's status in the real world, and one's future (10). In other words, the major depressive disorder is a complex and heterogeneous illness with an etiopathogenesis that is based upon multiple factors that may act at different levels, e.g., psychological, biological, genetic, and social (11).

Two current reference classifications provide a clinical description of depression: the Diagnostic and Statistical Manual of Mental Disorders, Fourth Edition (DSM-IV) and the International Classification of Diseases, 10th Edition (ICD-10).

According to the DSM-IV, one of the principal forms of depression is major depressive disorder (MDD). For an appropriate diagnosis, five or more of the following 10 DSM-IV symptoms must be exhibited:

- Depressive mood present continuously for a minimum 2-week period prevalent every day and a larger part of the day
- Pronounced elevated emotional psychomotor activity in children and teenagers
- Diminished ability to feel pleasure and rejoice
- Loss or gain of weight against a marked appetite alteration
- Sleep disturbances: insomnia at night and daytime sleepiness
- Objectively registered psychomotor agitation or motor retardation
- Feeling of weakness, loss of energy, marked fatigue even after minimal effort
- Lowered self-esteem and feeling of worthlessness, loss of self-confidence, ungrounded self-accusation to the extent of delirium
- Diminished ability to think or concentrate, mental slowness, lack of resolution
- Thoughts or actions leading to self-injurious or suicidal ideation.

The abovementioned symptoms must be present continuously for at least 2 weeks and cause disturbance of a person's normal vital activities.

In terms of the manifestation of depressive symptoms, the contemporary ICD-10 classification identifies a number of clinical variants of depression, which are classified according to their severity, the presence of psychiatric symptoms, and recurrence of DDs. Episodes of moderate depression (four or more symptoms exhibited; F32.1) and severe depression without psychotic symptoms (commonly with a number of symptoms, usually with lowered self-estimation and suicidal thoughts and attempts; F32.2), and a recurrent (repeated) DD of moderate or severe degree (F33.2–F33.3).

It is important to note that the main classifications of DDs have some differences and that this must be considered when analyzing the experimental data in studies of depression in humans. It is possible that some of the differences in results between studies will be related to the use of different samples of depressed patients who have been diagnosed according to different clinical criteria. Moreover, in our opinion, it is necessary to distinguish exo- and endogenous DD and analyze this type of DD separately. Exogenous, or reactive, depression is usually triggered by some situational stress, as losing a job, the loss of a member of the family, divorce, or relationship difficulties. As opposed to endogenous depression, exogenous is environmentally caused, and associated with anxiety and mood reactivity, and highly sensitive to psychosocial stressors. Endogenous or melancholic depression is a form of DD unrelated to any pronounced exogenous factors (primary severe somatic illness, illness in a close relative, perceived social problems. In exogenous DD can clearly determine the causes of this disease.

## Major hypotheses of pathogenesis of depression: from clinical and biochemical data to candidate genes of depression

### The monoamine theory

The monoamine hypothesis of depression, the first theory historically, was proposed by Joseph Schildkraut in the 1960s. This theory was based on the successful use of iproniazid (monoamine oxidase inhibitor) (12–14) and imipramine (reuptake inhibitor of monoamine neuromediators) for depression (15, 16). As proposed by this theory, insufficiency of monoamine neuromediators (serotonin, norepinephrine, dopamine) in definite structures of the central nervous system (CNS) may lead to the development of depression. Detailed analysis of the mechanism of action of these preparations and of later designed tricyclic antidepressants and reuptake inhibitors of monoamine neuromediators confirmed the important role of the imbalance and insufficiency of neuromediators in DDs (17–20). According to the monoamine theory, the synthesis, vesicular transport, and receptors of monoamine neuromediators play an important role in the development of DDs. As a result, the first genetic studies focused on identifying and analyzing polymorphisms in genes associated with serotonin, noradrenalin, and dopamine neurotransmission.

Most studies have analyzed *SLC6A4* (previously known as *SERT*), which encodes the serotonin transporter that is responsible for the reuptake of serotonin (5-HTT) from the synaptic cleft to the presynaptic neuron and thus plays a role in maintenance of the serotonin level in the presynaptic region. Interest in this transporter also arises from the observation that inhibitors of neural serotonin reuptake are used widely in psychiatry for the treatment of depression, anxiety, and other conditions.

The serotonin transporter is encoded by the solute carrier family 6 member 4 gene (*SLC6A4*) located on chromosome 17q11.1–17q12 (21). In the promotor region of *SLC6A4*, a 5-HTTLPR (5-*h*ydroxy*t*ryptamine *t*ransporter-*l*inked *p*olymorphic *r*egion) polymorphism was shown to be associated with the availability (the long L allele) or absence (the short S allele) of the 44 bp fragment (22). The L allele bears 16 GC-rich repeated elements of 20–23 bp, whereas the S allele carries 14 similar repeated units that result from the deletion of the region from the 6th to 8th repeated elements (23). *In vitro* studies have shown that the S allele is associated with a lower expression level of *SLC6A4* mRNA and lower serotonin transporter expression on membranes and, as a consequence, with a lower ability for serotonin reuptake compared with the L allele (23, 24). A number of other rare variants of 5-HTTLPR polymorphism, which contained 15, 19, and >20 repeats, were later reported (25).

In 2006, the single-nucleotide polymorphism (SNP) rs25531 (A → G) near the 5-HTTLPR polymorphism region was revealed by Hu and coauthors. This polymorphism appears to show linkage disequilibrium with 5-HTTLPR, and the G variant is only found in the L allele carriers. The A → G substitution evokes the appearance of the L_G_ allele, the functional analog of the 5-HTTLPR S allele (14, 26). This is because the A → G substitution creates a strong AP2–DNA-binding site (TFBS) which, in turn, suppresses the transcription of *SLC6A4* in LG allele carriers (13, 14). It appears that up to 15% of the individuals included in previous studies as L allele carriers should have been functionally classified as S allele carriers. This error may have distorted the results and created false-positive or false-negative evidence. Moreover, the situation appears to be more complicated and clearly demonstrates possible problems in interpreting the results of association analysis of individual polymorphic markers inside candidate genes.

In 2008, another SNP, rs25532 (C → T) also localized near 5-HTTLPR, was identified in the promotor region of *SLC6A4*. This SNP changes the activity of the 5-HTTLPR/rs2553 combination of polymorphisms. For instance, the L_AC_ allele (the combination of the L allele at the 5-HTTLPR polymorphism with the A and C alleles at the rs25531 and rs25532 polymorphisms) is a variant that ensures a high level of *SLC6A4* expression (27). Further studies revealed additional SNPs, with functionally significant changes, such as G56A in exon 2 and 1425V in exon 9. The 1425V mutation is located in the transmembrane area of 5-HTT, which is important for the formation of the secondary structure of this hydrophobic domain.

A polymorphic region comprising three alleles, Stin2.9, Stin2.10, and Stin2.12, was discovered in intron 2 of *SLC6A4*. This variable number tandem repeat polymorphism increases the expression in proportion to the number of repeated copies of the 16/17 bp element (12 > 10 > 9), as determined in the embryonic brain and in human JAR cells (28). Stin2 alleles respond differently to transcription factors YB-1 and CTCF which, in turn, can be regulated by lithium chloride, which is prescribed for the treatment of bipolar disorders (28, 29).

The structure of *SLC6A4* may be far more complicated. Recent publications have reported that the expression of this gene is modulated by microRNA mir-16 binding sites in the 3′-nontranslating region of the gene (30). Therefore, polymorphisms localized within or near microRNA binding sites may be able to exert a strong effect on *SLC6A4* expression and, consequently, on 5-HTT functions.

It is possible that the abovementioned complexity of *SLC5A4* organization may be one reason for the conflicting results obtained by analyses of the association of this gene's polymorphic variants (primarily in the analysis of the L/S polymorphism of the 5-HTTLPR repeat) with the onset of depression. Meta-analyses of these studies allow no final conclusion to be drawn about the role of this polymorphism in the development of depression. For instance, the meta-analysis conducted by Lopez-Leon et al. (31) disclosed an elevated risk of DDs in S allele carriers, whereas no similar association was found in carriers of other alleles (32–34). The latest meta-analysis of the results of 23 original studies has shown that the S allele raises the risk of DDs; the risk of depression in S allele carriers is increased 1.14-fold (CI: 1.05–1.24). Nevertheless, the high level of heterogeneity of the data included in this meta-analysis should be noted. In all analyzed models, the association *p*-value does not reach 0.05. This may be due to the inclusion in the meta-analysis of studies of different size samples, including samples comprising fewer than 50 people (35).

In the context of the monoamine theory of DD development, analysis of a large number of candidate genes has been performed. They are, in particular, receptor genes for dopamine (*DRD3, DRD4*) and serotonin (*HTR1A, HTR2A, HTR1B, HTR2C*); genes for noradrenalin (*SLC6A2*) and dopamine (*SLC6A3*); genes for the enzymes monoamine oxidase A (*MAOA*), tyrosine hydroxylase (*TH*), tryptophan hydroxylase 1 (*TPH1*), catechol-o-methyl transferase (*COMT*); and the piccolo presynaptic cytomatrix protein (*PCLO*). For each of these genes, polymorphic variants were identified that were associated with point mutations or tandem repeat polymorphisms. These polymorphisms were analyzed in samples from patients of different ethnicity with DD. As for *SLC6A4*, different studies have produced conflicting results, and it seems reasonable not to analyze the results of individual studies but to consider only the meta-analyses that have shown the existence of associations between definite variants of the genes and DD development.

One of the first large-scale meta-analyses of genetic case–control research on DDs was conducted in 2008 by Lopez-Leon and coauthors. The final analysis focused on 20 polymorphisms in 18 genes. The pooled odds ratios (ORs) with 95% confidence intervals (CIs) were calculated. Among the genes of the monoaminergic system, statistically reliable associations were found for *SLC6A4* and *SLC6A3* (31).

In another meta-analysis, Gatt et al. (36) attempted to identify genes associated with DD and common genes shared by the five severe psychiatric disorders: MDD, anxiety (including panic disorders), schizophrenia, bipolar disorder, the attention deficit–hyperactivity syndrome (36). Table 1 displays the data for the genes whose products are involved in monoaminergic neurotransmission.

**Table 1:** Associations shown in meta-analyses between DDs and polymorphic variants of the genes linked to the exchange of monoamine neuromediators^*^.

	**Gene**	**Polymorphism**	**Total number of studies included in meta analyses**	**OR**	**Risk allele**
*DRD4*	Dopamine receptor D4	48-bp VNTR	5	1.73	2-repeat
*HTR1A*	5-hydroxytryptamine receptor 1A	rs6295 (C1019G)	9	0.82	*G*- allele
*MAOA*	Monoamine oxidase A	VNTR polymorphism in promotor region	9	1.23	*L*- allele
*PCLO*	Piccolo presynaptic cytomatrix protein	rs2522833	3	NR	*C*- allele
*SLC6A3* or *DAT1*	Solute carrier family 6 member 3	40-bp VNTR	3	2.06	9/10 - genotype
*SLC6A4* or *5-HTT*	Solute carrier family 6 member 4	44-bp Ins/Del (5-HTTLPR)	24	1.11–1.23^*^	*S*- allele
*TPH2*	Tryptophan hydroxylase 2	rs4570625	6	0.77–0.83	*G*- allele
		rs11178997	4	0.75	*T*- allele
		rs17110747	5	0.79 - 0.84	*G*- allele

* *According to Gatt and coauthors with modifications (36)*.

It appears that polymorphic variants of the genes that are in some way associated with the monoamine theory of DD pathogenesis can influence the risk of developing depression. However, this influence is not large and cannot be currently regarded as unambiguously proven because of conflicting results of both individual studies and meta-analyses.

### Stress as a cause of depressive disorders

**Figure 1:**
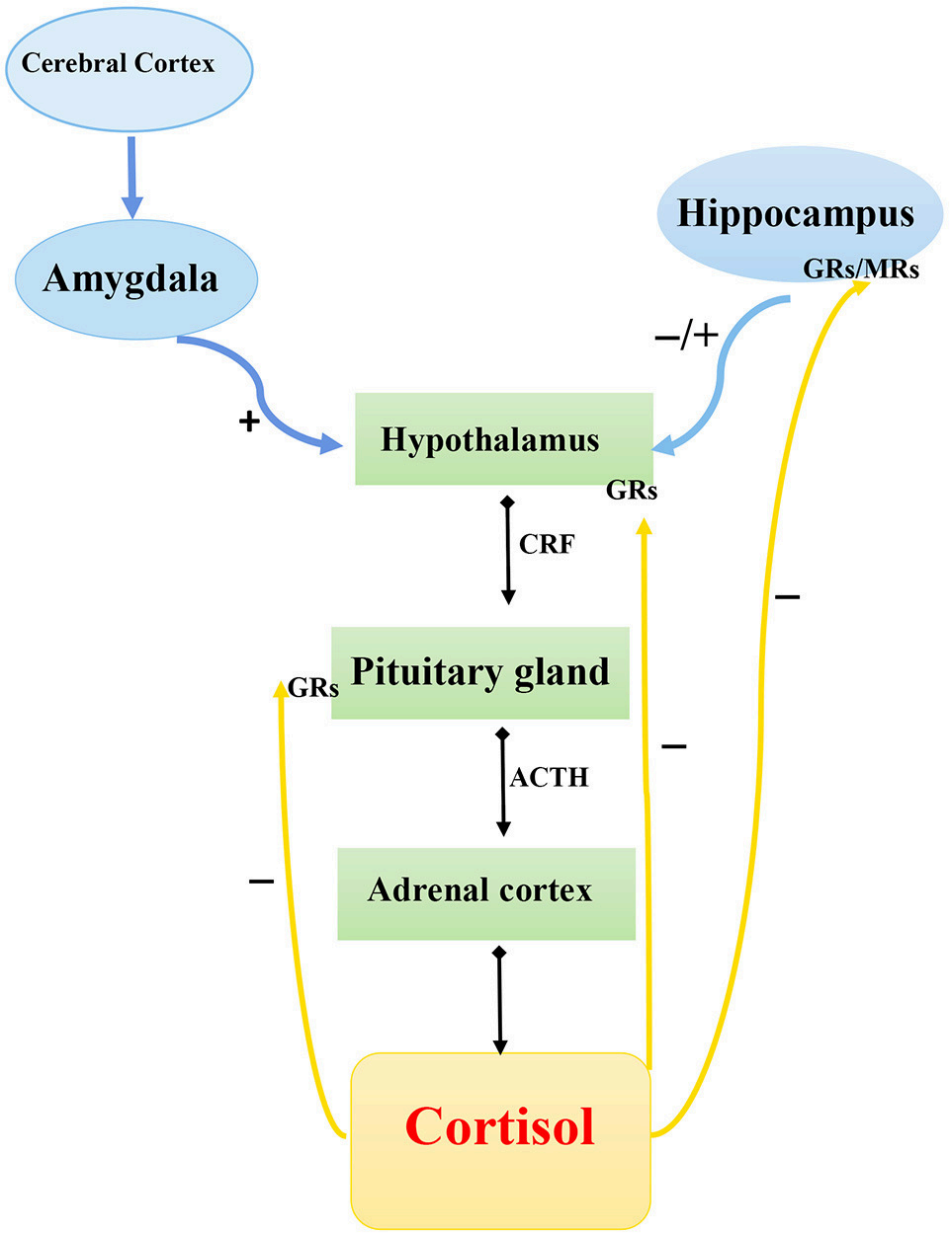
Schematic diagram of hypothalamic–pituitary–adrenal axis. CRF, corticotropin-releasing factor; ACTH, adrenocorticotropic hormone; GRs, glucocorticoid receptor; MRs, mineralocorticoid receptors. 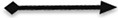, Secretion; 
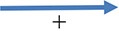
, stimulation; 
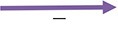
, inhibition.

Chronic stress and stressful life events early in life are strong proximal predictors of the onset of depression. Although the response to stress implies stability or maintenance of homeostasis, long-time activation of the stress system can cause harmful or even fatal consequences by elevating the risk of obesity, heart diseases, depression, and other disorders (37). The Hypothalamic–pituitary–adrenal axis and its three main components—hypothalamic neurosecretory cells, pituitary gland, and adrenal cortex—are responsible for adaptation to changed environmental conditions and for mobilization of the organism's reserves during exposure to stress of different etiologies. The HPA system operates in the following way (Figure 1). In response to a stressor, neurons in the hypothalamic paraventricular nuclei secrete corticotropin-releasing hormone (CRH), which exerts its action on the hypophysis to initiate the release into the blood circulation of adrenocorticotropic hormone (ACTH), which stimulates the release of corticosteroids, particularly cortisol, from the adrenal cortex. The final hormonal product of the HPA axis, cortisol, binds to mineralocorticoid receptors (type 1) and glucocorticoid receptors (type 2) to form hormone–receptor complexes, which are then transported into the cell nucleus where they interact with specific DNA regions, the glucocorticoid-response elements, to activate the expression of hormone-dependent genes (38).

The “stress-induced” theory of DD onset is based on the assumption that hyperactivity of the HPA system may be an important mechanism underlying the development of depression after exposure to stress. A number of examples of abnormal functioning of the HPA system during depression favor this hypothesis. First, stressful events during one's life are the strongest factors that can initiate depression onset (39, 40). Second, depressed patients frequently show elevated cortisol levels (the human endogenous glucocorticoid) in plasma, urine, and cerebrospinal fluid and corticotropin (ACTH) level in plasma (41). Depressed patients also exhibit increased size of the hypophysis and suprarenal glands (42) or decreased function of corticosteroid receptors (43). Excessive activation of the HPA axis is observed in 50% of depressed people, and continuous administration of antidepressants helps to attenuate this activation (44).

A large cohort of genes is likely to be involved in the normal functioning of the HPA axis, but only some of these genes have been actively investigated in the context of DDs. The genes most likely to be involved in DDs are those encoding the targets of cortisol and other glucocorticoid hormones secreted during stress. Polymorphic variants of these genes were analyzed in case-control studies and in some cases, associations between these variants and development of DD were shown. For instance, associations between DD onset and polymorphic sites of the genes coding for the GR (*NR3C1*) and mineralocorticoid receptor (MCR; *NR3C2*) have been reported (45–47). Moreover, *postmortem* studies by Klok et al. (46) have shown that MCR mRNA expression is reduced in the hippocampus in depressed patients (46).

Associations with DD were also shown for genes *CRHR1* and *CRHR2*, which encode CRH receptors (48). Lui et al. (48) reported significant associations between depression and the SNV rs242939 in the *CRHR1* gene. These authors also showed that the haplotype formed by G–G–T alleles of the rs1876828, rs242939, and rs242941 was most often represented in patients with MDD compared with controls (48). Szczepankiewicz et al. (49) found associations between DD and the SNVs rs4076452 and rs16940655 of *CRHR1* gene (49). Xiao et al. (50) also reported associations between the rs242939 polymorphism of *CRHR1* gene and recurrent depression (50).

We note that some of the associations described earlier were included in the meta-analyses by Lopez-Leon et al. (31) or Gatt et al. (36). These authors did not find any reliable associations between DDs and polymorphisms in the genes involved in the functioning and regulation of the HPA axis (31, 36).

### Disturbance of neurogenesis and neuroplasticity

A large body of experimental data has recently provided evidence of a link between the development of depression with disturbance of normal neurogenesis during brain ontogenetic development and decreased neurogenesis of the adult brain. These effects are thought to be caused by metabolism disturbance of neurotrophic factors, primarily the brain-derived neurotrophic factor (BDNF) in nervous tissue (51–57).

BDNF is abundantly expressed in the adult brain's limbic structures. Some data have shown a connection between the BDNF-mediated signaling pathway and the functioning of serotoninergic neurons. For example, BDNF maintains the survivability and differentiation of serotoninergic neurons, and serotoninergic transmission exerts a strong influence on BDNF expression (58–60).

A functional missense polymorphism rs6265 (GI96A) was described in *BDNF* that is associated with the substitution of methionine (Met) with valine (Val) in codon 66 (Val66Met). The Met allele was shown to cause disturbed maturation of the protein and to be associated with decreased BDNF activity (61), which might be caused by two different mechanisms: the infective transport of the mutant protein over the regulatory secretory pathway and the defective transport of *BDNF* mRNA to dendrites (62). Val66Met is a frequent polymorphism whose frequency of alleles is determined by ethnicity. Met allele frequency is 25–32% in European populations and reaches 40–50% in Asian populations (63).

Some studies have reported an association of the Val66Met polymorphism in *BDNF* with DD onset (64–66), but different authors attribute the risk of DD to the effect of different allelic variants. For instance, Frielingsdorf et al. (66) showed that Met allele homozygotes are at significantly increased risk of MDD (66), whereas Ribeiro et al. (65) defined the Val allele as an allele for the risk for depression (65). This inconsistency motivated the publication of a meta-analysis that combined the results of 14 original studies, but this analysis did not confirm an association between the polymorphic variant in *BDNF* with DDs (63, 67). The effect of this polymorphism may have been observed only in interaction with other polymorphic systems, such as with 5-HTTLPR polymorphisms or after exposure to severe stress (68, 69).

### The cytokine theory

The brain was earlier thought to be an “immune-privileged” organ that is protected from circulating immune cells by the blood–brain barrier. It is now well known that immune system cells can infiltrate into nervous tissue. Pro- and anti-inflammatory signals can be transmitted to nervous system from peripheral areas. Also, cytokines and their receptors can be produced in the CNS by astrocytes, microglia (70) and, in some cases, neurons. These molecules are believed to participate in the processes of neuronal development, plasticity, synaptogenesis, and tissue repair.

The hypothesis of bidirectional communication between the immune system and the CNS was suggested in the 1990s. According to this hypothesis, the immune system can interact with the CNS as well as being involved in neuropathological processes. In 1999, Maes *M*. proposed the inflammatory response system (IRS) model of depression, which claimed that the occurrence of depression depends on activation of the IRS. According to this model, depression can be regarded as a psychoneuroimmunological disease in which peripheral activation of the immune system through the release of anti-inflammatory cytokines can cause the various behavioral, neuroendocrine, and neurochemical changes observed in this disorder (71). This theory was later extended and is now referred to as the “cytokine theory.”

Cytokines constitute a heterogeneous class of mediator molecules produced as regulators of the immune response by immunocompetent cells such as lymphocytes and microphages. Cytokines can be classified into two groups: proinflammatory and anti-inflammatory cytokines. Proinflammatory cytokines are either immediately or indirectly involved in the inflammatory process: interleukin 1 (IL-1), IL-2, IL-6, and IL-12; interferon γ (IFNγ); and tumor necrosis factor α (TNFα). Anti-inflammatory cytokines include IL-4, IL-10, and IL-13, which suppress the immune response and thereby prevent both cell activation and the production of proinflammatory molecules. Some cytokines, such as IL-8, exert both the pro- and anti-inflammatory functions, according to their concentration.

Immunological changes during depression and psychiatric side effects caused by the use of cytokines in the treatments for hepatitis and cancer provide evidence in favor of this theory. For instance, stressors increase the expression of proinflammatory cytokines such as IL-1β, TNFα, and IL-6) in blood and brain (72, 73). DD patients display increased granulocyte and macrophage counts in peripheral blood (74).

In addition to associations with the depression, associations between inflammatory markers and some symptoms, such as apathy, cognitive dysfunction (75), and impaired sleep (76), have been reported. For instance, sleep impairment in depressed patients is associated with increased levels of IL-6 and the soluble forms of intercellular adhesion type 1 molecules in plasma (76) and with the activation in blood cells of nuclear factor-κB (NF-κB), the principal transcription factor involved in initiation of the inflammatory response (77). In other studies, about 50% of the patients chronically administered IFNα developed symptoms of depression, which further supports the role of inflammation in DD pathogenesis (78, 79).

We note, however, that the average cytokine levels in the plasma are elevated only slightly in DD patients compared with healthy people and that these levels are sometimes close to the physiological norm. In autoimmune or infectious diseases, the cytokine levels increase considerably more. Therefore, DD is not considered as a typical autoimmune disease (74).

*IL1B* is located at locus 2q14.1 on chromosome 2. Several SNPs have been reported for this gene, and two—rs1143627 (−31T/C) and rs16944 (−511C/T)—are associated with DD. Some studies have reported an association between rs16944 (−511C/T) and depression onset and with a positive response to treatment with some antidepressant medications (80–83). Borkowska et al. (81) reported a positive association with recurrent depression (*P* = 0.064) for the polymorphic haplotype comprising the rs1143627 SNP C allele and the rs16944 SNP T allele (81). By contrast, Yu et al. (82) did not confirm an association of the rs14944 SNP with major depression, although the severity of depression symptoms was elevated in allele C homozygotes (82). Similarly, Hwang et al. (84) failed to find any reliable associations of rs16944 SNP with senile depression or the severity of depression symptoms (84).

The SNPs rs1143627 (−31T/C) and rs16944 (−511C/T) are found in the promoter region of the gene and can affect the expression of this gene, which influences the IL-1β level. Chen et al. (85) showed that SNP rs1143627 localizes in the TATA box area in the IL1B promoter and that the T allele of this polymorphism is associated with increased production of IL-1β (85). However, other investigations have failed to find any reliable associations of SNP rs1143627 with IL-1β production *in vitro*, although one study found an association between elevated IL-1β expression *in vivo* and the C allele (86). Data concerning the influence of rs16944 (−511C/T) on *IL1B* expression are also conflicting. For instance, Hall et al. (87) showed that this SNP influences the expression directly (87). On the other hand, some data support the concept that the −511C/T polymorphism influences the expression of the gene only when acting in concert with the −31T/polymorphism (85).

Therefore, the current data concerning an association of polymorphic variants of the *IL1B* promoter area do not allow any unambiguous conclusions to be drawn about the role of this gene in the development of depression.

Another actively studied gene, *IL6*, encodes the proinflammatory cytokine IL-6 and is located at the 7p15.3 locus. The functional polymorphism rs1800795 (174*G/*C) is located in the gene's promoter region and influences gene expression both at the mRNA and protein levels. The IL-6 level is lower in *C* allele carriers under conditions of immune system activation (88). No association of this polymorphism with major depression, depression in children, or postbrain stroke depression has been found (89–91). However, this polymorphism has been reported to affect the progression of depressive symptoms in hepatitis C patients administered the immunomodulators IFNα and ribavirin (92). Another interesting observation in that study was the interaction between the 5-HTTLPR polymorphism in the serotonin transport gene (*SLC6A4*) and rs1800795. A “protective” effect of the 5-HTTLPR polymorphism was observed only in the presence of the low-expressing genotype for *IL6* (*CC*). Consequently, in the genetics of depression, the transition from the analysis of individual polymorphic variants to the analysis of their combinations including two, three, or more loci seems to be very important.

### The circadian rhythm theory

Circadian rhythms oscillate with ~24-h periodicity and are responsible for regulating a wide variety of physiological and behavioral processes. Endogenous cyclic oscillations are regulated in humans and other mammals by the circadian pacemaker—the suprachiasmatic nucleus (SCN) neurons of the anterior hypothalamus (93). The circadian pacemaker can change its pattern so that the circadian rhythm may be advanced, delayed, or remain constant in various pathological states or when affected by different pharmacological preparations and hormones; for instance, melatonin regulates the function of the biological clock through melatoninergic receptors residing in the hypothalamic SCN (94). At the cellular level, the “molecular clock” refers to a network of “clock” genes, which are transcriptional regulators organized in a feedback-sustained transcription–translation network. This mechanism helps maintain the rhythmic expression of target genes during a 24-h cycle (95). The basis of the molecular clock is the negative feedback loop, in which the expression of PER and CRY proteins is inhibited by their interactions with the transcriptional factors CLOCK and BMAL1 and by blocking their binding to E-box regulatory elements in the promoters of the genes for PER and CRY protein family members. Posttranslational modifications of the molecular clock system components by signal molecules such as casein kinase δ/ε and glycogen synthase kinase 3-beta play important roles in the maintenance of circadian rhythms (96).

Sleep disorders (early awakening, insomnia, and inability to resume sleep after waking) are observed in 80–90% of patients with depression, and insomnia is regarded as a risk factor for the onset of depression (97). Therefore, disturbance of the normal functioning of circadian system proteins may play a role in DDs. Additional evidence for the role of circadian rhythm genes in DD onset was provided by studies of two familial syndromes of sleep disturbance: familial advanced sleep-phase syndrome and delayed sleep-phase syndrome (DSPS). Both syndromes are caused by mutations in the genes *PER2, CKie, PER3*, and *CLOCK*, which encode circadian system proteins (98). People with these mutations frequently experience depressive symptoms. In addition, people with a history of depression have elevated expression of the circadian system genes *CLOCK, PER1*, and *BMAL1* compared with healthy volunteers (99). Case–control studies have shown associations between depression onset and polymorphic variants of the genes encoding circadian system proteins, including *BMAL1, CLOCK, NPAS2, PER3, CRY1*, and *TIMELESS*. However, none of these genes was confirmed in the meta-analysis (100).

### Other candidate genes

Investigations of the candidate genes selected according to the principles of DD etiopathogenesis theories have identified only five genes whose polymorphic variants are reliably associated with DD onset according to the results of meta-analyses (Table 1). However, all of these genes have been discussed only in terms of the monoamine theory of depression.

The association research is not confined solely to the DD-related candidate genes studied in terms of DD etiopathogenesis theories. Other genes have also been actively studied, primarily those analyzed for other neurological and psychiatric disorders. Moreover, these studies have revealed a large amount of reliable associations that have passed through testing in large meta-analyzes (Table 2). These association studies have revealed genes encoding functionally diverse proteins, from chondroitin sulfate biosynthesis enzymes to the key enzyme of the renin–angiotensin system (RAS). On the one hand, this may reflect the limited understanding of DD etiopathogenesis, although the currently known associations suggest new hypotheses. On the other hand, many of the associations revealed are in some way linked to neurogenesis and neuroplasticity processes, and DDs may be regarded as disorders linked to disturbance of neural tissue ontogenesis.

**Table 2:** Association shown by meta-analyses between DD and polymorphic variants of genes not linked with the general hypotheses of DD etiopathogenesis.

**Gene**	**Function**	**Polymorphism**	**Total number of studies**	**OR**	**Risk allele**
*ACE* (angiotensin I converting enzyme)	Encodes the key enzyme of the RAS; it catalyzes conversion of angiotensin I to angiotensin II and is involved in blood pressure control	Ins/Del	15	1.18–1.20	DD vs. II/ID genotype
*APOE* (apolipoprotein E)	The protein encoded by this gene forms part of chylomicrons and very low-density lipoproteins; it plays an important role in lipid and cholesterol exchange, and activates lipoprotein lipase and lecithin choline acyltransferase	ε2/3/4	7	0.51	ε3 vs. ε2
*MTHFR* (methylenetetrahydrofolate reductase)	The protein encoded by this gene plays a key role in folic acid metabolism by converting 5,10-methylenetetrahydrofolate, a coenzyme involved in homocysteine remethylation	rs1801133	10	1.14–1.20	*T*-allele
*CHST11* (carbohydrate sulfotransferase 11)	The protein encoded by this gene plays a key role in chondroitin sulfate biosynthesis	rs1344677	2	1.32	*T*-allele
*PTPRR* (protein tyrosine phosphatase, receptor type R)	The protein encoded by this gene is a member of the protein tyrosine phosphatase family, which is involved in the regulation of many cellular processes, such as cell growth, differentiation, mitosis, and oncogenic transformation.	rs4760933	2	0.60	*G*-allele
*ADCY9* (adenylate cyclase 9)	Encodes the enzyme that catalyzes the conversion of adenosine monophosphate (AMP) to cyclic AMP	rs2239307	2	0.65	*C*-allele
*ITPR1* (inositol 1,4,5-trisphosphate receptor type 1)	Encodes an intracellular receptor for inositol 1,4,5-trisphosphate, which mediates release of calcium from the endoplasmic reticulum	rs9311395	2	0.78	*G*-allele
*DNAJB2* (DnaJ heat shock protein family (Hsp40) member B2)	Encodes a neuronal chaperone that may help to protect against the development of neurodegenerative processes	rs7596956	2	0.72	*C*-allele
*EHD3* (EH domain containing 3)	The protein encoded by this gene controls cell membrane reorganization and endocytosis processes through the transport of endosomes to cell membranes and endosome recycling in the Golgi complex	rs590557	2	0.65	*G*-allele
*FREM3* (FRAS1 related extracellular matrix 3)	Encodes an extracellular matrix protein that may play a role in cell adhesion	rs7676614	2	1.32	*A*-allele
*GNB3* (G protein subunit beta 3)	Encodes a G-protein that acts as a modulator and switch in transmembrane signaling systems and exhibits GTPase activity	rs5443	3	1.38	*T*-allele
*PHACTR3* (phosphatase and actin regulator 3)	The protein encoded by this gene is associated with the nuclear scaffold in proliferating cells and can bind to actin and the phosphatase 1 catalytic subunit	rs8122984	2	0.76	*G*-allele
*HS6ST3* (heparan sulfate 6-O-sulfotransferase 3)	The protein encoded by this gene modifies heparin sulfate and contributes to the formation of structures needed for heparin sulfate interaction with different proteins; such interactions are involved in cell proliferation, differentiation, adhesion, inflammation, and other processes	rs17767562	2	0.76	*C*-allele
*KLHL29* (kelch like family member 29)	Unknown	rs1653765	2	0.69	*G*-allele
*LHFPL2* (lipoma HMGIC fusion partner-like 2)	Unknown	rs12651937	2	0.76	*C*-allele
*SLC25A21* (solute carrier family 25 member 21)	Encodes a protein that ensures oxodicarboxylate transport across the inner mitochondrial membrane	rs17105696	2	0.51	*G*-allele
*UGT2A1* (UDP glucuronosyltransferase family 2 member A1 complex locus)	Encodes a protein that takes part in phase II detoxification of xenobiotics and catalyzes the conjugation of lipophilic substrates with glucuronic acid	rs6832167	2	0.68	*G*-allele
*VGLL4* (vestigial like family member 4)	Encodes a coactivator of transcription factors	rs6781822	2	1.35	*T*-allele

*According to Gatt and coauthors with modifications (36)*.

Associations between DD and polymorphic variants of the genes angiotensin-converting enzyme (*ACE*), apolipoprotein E (*APOE*), and methylenetetrahydrofolate reductase (*MTHFR*) are of special interest. These genes have been discussed in the context of another theory of DD etiopathogenesis—the vascular theory—which postulates that DD occurs because of disturbance in the blood supply to neural tissue. According to this theory, vascular disorders can cause DD as well as other mental illnesses such as schizophrenia and manic–depressive psychosis. It is possible that there is a continuum of these diseases, which are separated by only a thin line; for example, some clinical subtypes of depression are characterized by marked psychotic symptoms (F32.3 in ICD-10). Presumably, there may be a complex link between genetic variants and the occurrence of various pathologies along the continuum. For example, genetically determined vascular disorders provoke increased risk for different mental disorders. Progression of a particular disease (e.g., schizophrenia, depression, manic–depressive psychosis) may be determined by other genetic factors, such as those associated with neuromediator functions.

## Genome-wide association analysis of depressive disorder

As noted above, >20 genes have been associated with DD onset and confirmed by meta-analyses. In most cases, these associations involve genes that are not directly linked to the general theories of depression ethnopathogenesis. Association studies appeared to be connected with transition to genome-wide methods of association analysis without any suggestions about the genetic risk factors of depression.

In the first stage of GWASs, families with members that have experienced multiple depression events, severe course of the disorder, or an early age at its clinical onset were analyzed with special interest in patients with rare monogenic forms of depression. The results of these studies are summarized in Table 3. These studies have reported associations with extended genomic regions (even as long as full-length chromosomes), and the identification of the candidate genes seems to be provisional. This identification mainly based on the DD candidate genes mapped earlier in these genome regions.

**Table 3:** Mapping the loci associated with predisposition to different forms of depression in family studies using SNP panels of DNA markers.

**Reference**	**Clinical phenotype**	**Chromosome**	**Candidate gene**
(101)	Recurrent depression with early onset	15q25.3–26.2	*NTRK3* (neurotrophin 3 receptor)
(102)	Recurrent depression, depression-predominant bipolar disorder	12q23	NA
(103)	Depression with early (31 years of age) onset; depression with anxiety	chromosomes 3centr, 7p and 18q	NA
(104)	Recurrent depression without symptoms of bipolar disorder	1p36, 12q23.3-q24.11 and 13q31.1-q31.3	NA
(105)	Depressive disorder	chromosomes 17 and 8	*SLC6A4* (solute carrier family 6 member 4)

GWASs have been used increasingly in the past decade to identify loci that control complex traits. In this analysis, as many as hundreds of thousands to several millions of SNPs distributed over the whole genome are identified in groups of persons having a particular trait of interest. Analysis of the genotype–phenotype associations makes it possible to establish a link between the allelic variant in some particular region of the genome with the trait studied. The principal difference between GWASs and candidate gene studies using the case–control method is that there is no preliminary hypothesis to explain the contribution of polymorphic variants of genes to the development of a pathology of interest. However, for a study to achieve statistically significant results, its algorithm requires very large samples of both patients and healthy persons. It can be extremely difficult to achieve clinical homogeneity in very large samples, especially when studying psychiatric diseases because there is always a subjectivity factor affecting the diagnostic accuracy in the relevant international classifications with almost no instrumental methods for assessing the patient's condition.

A number of studies have searched for loci associated with MDD or individual symptoms of depression. The results are summarized in Table 4. This table focuses mainly on those studies that analyzed primarily the risk of depression as a disease and not the endophenotypes (e.g., clinical onset age, severity of particular symptoms, patients' responses to therapy). As well, Table 4 includes the most statistically significant results from the analyzed articles.

**Table 4:** Genome-wide association studies of major depressive disorders (MDDs) and recurrent depressive disorders (RDDs).

**Reference**	**Clinical phenotype**	**DNA-marker with the smallest P-value**	***P*-value**	**Gene near SNP**	**Gene function, metabolic pathway**
(106)	MDD	rs2715148	7.7 × 10^−7^	*PCLO* (piccolo presynaptic cytomatrix protein)	The protein encoded by this gene is part of the presynaptic cytoskeletal matrix involved in the formation of active synaptic zones and transport of synaptic vesicles
(107)	RDD	rs4238010	5.80 × 10^−6^	*CCND2 (*cyclin D2)	The protein encoded by this gene is involved in control of cell cycle regulation (Gl/S transition) in complex with CDK4 or CDK6 kinases
(108)	RDD	rs9416742 rs999845	1.30 × 10^−7^ 3.1 × 10^−6^	*BICC1* (bicaudal C homolog 1)	Encodes an RNA-binding protein that is involved in gene expression regulation by modulating protein translation in embryogenesis
(109)	MDD	rs2765501 rs7713917	1.66 × 10^−7^ 5.87 × 10^−5^	*CD5L* (CD5 Molecule Like) Near gene *HOMER1* (homer scaffolding protein 1)	Encodes a protein that participates in regulation of the inflammatory response The protein encoded by this gene is a member of the HOMER scaffold protein family, which plays an important role in calcium signalization in many cell types
(110)	RDD, MDD	rs17077450	1.83 × 10^−7^	Near gene *DSEL* (dermatan sulfate epimerase-like)	The protein encoded by this gene is involved in metabolism of dermatan sulfate and chondroitin sulfate
(111)	RDD, MDD	rs12462886 rs110634	1.73 × 10^−6^ 6.78 × 10^−7^	No *ATP6V1B2* (ATPase H+ Transporting V1 Subunit B2)	Gene desert Encodes a protein that is a noncatalytic subunit of vacuolar ATPase complex VI
(112)	MDD	rs545843	5.53 × 10^−8^	*SLC6A15* (solute carrier family 6 member 15)	Encodes a protein that is a potassium-dependent transporter of uncharged amino acids that can play a role in the transport of neuromediator precursors in neurons
(113)	MDD	rs1558477 rs2522840	2.63 × 10^−7^ 4.38 × 10^−6^	*ADCYAP1R1 (*ADCYAP receptor type I) *PCLO* (Piccolo Presynaptic Cytomatrix Protein)	The protein encoded by this gene is a receptor for pituitary adenylate cyclase-activating protein 1, which is involved in adenylate cyclase activation The protein encoded by this gene is part of the presynaptic cytoskeletal matrix involved in the formation of active synaptic zones and transport of synaptic vesicles
(114)	MDD	rs11579964 rs7647854	4 × 10^−6^ 5 × 10^−6^	*NVL* (Nuclear VCP-Like) *C3or70* (chromosome 3 open reading frame 70)	Encodes the AAA–ATPase superfamily protein NVL, whose different protein isoforms have been localized to distinct regions of the nucleus and have different functional properties Unknown
(115)	DD symptoms	rs8020095 rs161645	3 × 10^−6^ 8 × 10^−8^	*GPHN* (gephyrin) *NUDT12* (Nudix Hydrolase 12)	Encodes the tubulin-binding protein gephyrin, which is involved in glycine receptor “anchorage” of the cytoskeleton; it is needed for the localization of GABA_A_ receptors in the postsynaptic membrane. Encodes a protein that regulates the concentration of individual nucleotides according to ambient conditions
(116)	MDD	rs8050326 rs11152166	3 × 10^−7^ 3 × 10^−6^	*IRF8* (Interferon Regulatory Factor 8) *CCBE1*(Collagen And Calcium Binding EGF Domains 1)	Encodes the transcriptional factor of Interferon Regulatory Factor Family (IRF), that regulates the expression of genes stimulated by type 1 IFNs Encodes a protein that participates in extracellular matrix remodeling
(117)	MDD with late onset age	rs7647854	5 × 10^−11^	*C3orf170* (chromosome 3 open reading frame 170)	Unknown
(118)	DD	rs10485715	8 × 10^−9^	*BMP2* (Bone Morphogenetic Protein 2)	Encodes a protein that is a secreted TGF-beta superfamily ligand that is important in bone and cartilaginous tissue formation
(119)	DD in hepatitis C patients	rs1863918	8 × 10^−8^	*ZNF354C* (Zinc Finger Protein 354C)	Encodes a protein that is a transcriptional factor that binds to 5′-CCACA-3′-type sequences
(120)	MDD\symptoms	rs9825823	7 × 10^−10^	*FHIT* (Fragile Histidine Triad)	Encodes a P1-P3-bis(5'-adenosyl) triphosphate hydrolase, an enzyme involved in the metabolism of purines
(121)	MDD	rs12552	6.1 × 10^−19^	*OLFM4* (olfactomedin 4)	Encodes a protein that is an antiapoptotic factor that promotes tumor growth;
		rs1432639	4.6 × 10^−15^	*NEGR1* (neuronal growth regulator 1)	Encodes a protein that serve as cell - adhesion molecules and regulate cellular processes as neurite outgrowth and synapse formation
		rs12129573	4.0 × 10^−12^	*LINC01360 (long intergenic non- protein coding RNA 1360)*	Unknown
		chr5_103942055_D	7.5 × 10^−12^	Unknown	Unknown
		rs8025231	2.4 x 10^−12^	Unknown	Unknown

The first GWAS of a large representative sample (1738 DD patients, 1802 controls) was reported by Sullivan et al. (106). In this study, no association with any of SNPs achieved the value of genome wide significance. The maximum significance was found for the rs2715148 (*p* = 7.7 × 10^−7^). Also, in this genomic region near *PCLO* gene 10 more SNPs were associated with DD with relatively low significance (*p* = 10^−5^-10–6). They were mapped to a 167 kb region where *PCLO* was located (106). PCLO protein localizes in the cytoplasmic matrix of the presynaptic active zone and plays a significant role in brain monoaminergic neurotransmission. A possible role of this region in depression onset was confirmed by Hek et al. (115), who showed an association between the rs2522833 SNP in *PCLO* and DD in a population-based study from the Netherlands (115). Aragam et al. (113) found a close statistically significant association between DD development for the rs2715148 SNP (*P* = 5.64 × 10^−7^) in *PCLO* in women (113). This study found another SNP in *LGSN* that was associated with DD occurrence in men (rs9352774, *P* = 2.26 × 10^−4^). This gene is actively expressed in the human crystalline lens and encodes a protein related to GS-I and, to a lesser degree, to GS-II glutamine synthetases. This protein may play a role in glutamate exchange in both the retina and the nervous system.

A role of glutamate in DD was found in a GWAS conducted by Rietschel et al. (109). They found an association between DD and the rs7713917 SNP (*P* = 5.87 × 10^−5^) located in a putative regulatory region of *HOMER1*, which encodes proteins involved in glutaminergic processes via interaction with the metabotropic glutamate receptors mGluR1 and mGluR5.

We reiterate that the associations discovered in most GWASs did not attain a genome-wide significance level, primarily because of the genetic architecture of complex traits predisposing to depression. Adjustments of the genome-wide significance level are very rigorous, and we believe that SNP markers with a probability value close to the genome-wide threshold level should also be considered.

Some studies have achieved a genome-wide significance level. Kohli et al. (112) were the first to report an association between DDs and the rs1545843 SNP in *SLC6A15* (solute carrier family 6, neutral amino acid transporter, member 15) in a recessive model of the effect of this polymorphism on the risk of DDs (112). This gene encodes the neutral amino acid transporter, and different rs1545843 alleles were shown to have different *SLC6A15* expression levels in the hippocampus of epileptic patients. The authors presented additional evidence to support the involvement of this association and showed that the presence of the risk allele correlated with lower *SLC6A15* expression in the hippocampus, smaller hippocampus volume, and neuronal integrity *in vivo*. Lower expression of *Slc6a15* was also observed in the hippocampus of mice with elevated chronic stress susceptibility.

Kohli et al. (112) reported abundant data in support of the association between *SLC6A15* and DD. However, subsequent GWAS disclosed no significant associations with this gene. The data obtained in GWASs are often not reproducible, and only one gene, *PCLO*, appeared to be associated with DD in two GWASs.

The Psychiatric Genomics Consortium (PGC) performed a meta-analysis of GWAS data. Unlike the conventional meta-analyses, which summarize the statistical data for each constituent analysis examined, the PGS study brought together and examined individual genotypic and phenotypic data from patients from different research centers. The PGS published the results of its genome-wide comparative analysis of 9240 samples collected from DD patients and 9519 samples from a control group of nine European populations (122). However, in the PGS analysis, none of SNPs identified in earlier studies achieved a genome-wide significance level. The SNPs with the most significant values were rs11579964 (*P* = 1.0 × 10^−7^), which mapped near *CNIH4, NVL*, and *WDR26*, and rs7647854 (*P* = 6.5 × 10^−7^), which mapped near *C3orf70* and *EHHADH*. A subsequent replicative study conducted using an independent sample (6783 patients with MDD and 50,695 controls) did not confirm the associations mentioned.

Therefore, no locus has been shown to be consistently associated with a DD at a whole-genome significance level. Associations shown in independent samples have also not been reproduced. This lack of significance and reproducibility may reflect the particular features of the GWAS methodology, which has focused on polymorphic sites with a high minor allele frequency (>5%) in the associative analysis. These frequent polymorphic variants themselves are probably not pathogenically essential, but there may be disequilibrium linkages with rare variants of genes associated with DD pathogenesis. These rare variants may be specific for different populations. As a result, any association between the disease and a frequent polymorphic site may be found in one sample and may reflect the disequilibrium linkage of this polymorphic site with a rare, pathogenically significant variant in that sample. However, the pathogenically significant site may be missing in another sample and, as a consequence, no association of frequent polymorphism with DD occurrence will be found. In addition, the important role of rare genomic variants (a frequency <1%) has been reported in association with other mental disorders, such as schizophrenia and autism (123, 124).

To overcome these problems, transition from the analysis of polymorphic DNA markers using microarrays to low-coverage DNA sequencing may provide a new direction for research to identify DD-associated genetic variants. The first study of this kind was conducted within the CONVERGE Project (125) and included genome sequencing with an average coverage of 1.7 × in >9000 Chinese females; 5000 females out of this group were patients with melancholic depression, which is recognized as a more severe form of depression. This study found two loci bearing an association at a 10^−8^ significance level: one on the 5′-side of *SIRT1* (SNP rs12415800) and the other in an *LHPP* intron (SNP rs35936514). This association was confirmed in an independent sample of melancholic Chinese women, and the significance values combined for the two samples were 2.53 × 10^−10^ for *SIRT1* and 6.45 × 10^−12^ for *LHPP*. It is important to note that both associated SNPs occur frequently (e.g., the minimal allele frequencies were 45.3 and 26.2%), yet neither is included in the microarrays used widely for SNP marker typing and, therefore, may have been ignored in earlier GWASs.

Further analysis of the data in this project showed that frequent SNPs accounted for 20–30% of the DD risk dispersion, which suggested that the heritability of DD is evenly distributed over all chromosomes with preferential localization of DD-associated SNPs in both the coding and the 3′-untranslated areas of genes. DD patients showed an elevated frequency of unique mutations in gene coding regions, primarily in the genes actively expressed in nervous tissue (126).

Importantly, this study included a specific ethnic group (Han Chinese), which is sufficiently homogeneous, and only females, who show a higher heritability level as mentioned above, with a severe form of DD. This design included a more rigorous approach to inclusion of samples and consideration of factors such as the patients' sex, clinical DD variation, clinical onset age, and other factors that can affect the risk of disease and its progression. However, these factors may exert no influence on the risk of DD development; for example, the clinical onset age was recently shown to not affect the association analysis results in the Chinese CONVERGE sample (127).

Another study also found that ethnicity was important (128). That study included a combined analysis of the results obtained in the CONVERGE investigation of Chinese and of studies conducted by the PGC in different European populations. These studies found that some SNPs influence the risk of DD onset in both ethnic groups mentioned but, at the same time, detected a set of SNPs specific to each ethnic group. The highest contribution of genetic factors in both ethnic groups was observed in females and in recurrently depressed patients.

Powers et al. (116) attempted to include environmental factors into GWASs (116). They included as a factor stress-provoking events when including case–control pairs of patients in the study—a method referred to as *propensity score matching*. This analysis allowed them to reduce the heterogeneity of the samples with regard to the stress factor and to compare DD patients and healthy controls exposed to similar stressors.

The genetic structure of depression appears to be extremely complicated and involves a large number of loci, which cause various phenotypic effects and display complex interlocus interactions. Studies of the genetic structure suggest the need for a transition from the analysis of individual SNPs to that of sets of SNPs and, finally, to include a polygenic risk score, as used in genetics research of schizophrenia (129).

To address similar problem, a strategy for studying gene networks created by uniting signals from numerous SNPs and subsequent functional analysis of the signaling and metabolic pathways have been used with success. This approach provides for an increase in the power of comparative analysis of weak signals from numerous loci. The study by Song et al. (130) is an example of such an analysis. On the basis of a GWAS of samples from European cohorts, the authors conducted a search and analysis of DD-linked SNPs and genes with these SNPs to discover signal pathways linking these genes to each other (130). Five resulting signal paths were found to play a role in DD pathogenesis. Three of them were claimed to be connected in some way with the negative regulation of gene expression (GO:0016481, GO:0045934, GO:0010629) and were related to some DD-associated SNPs: rs3213764 in *ATF7IP*; rs2301721 in *HOXA7*; rs6720481 in *LRRFIP1*; rs2229742 in *NRIP1*.

Okbay et al. (118) and Hyde et al. (131) offered an alternative approach for sampling (118, 131). To diagnose DDs, they compiled a questionnaire to be completed by the respondents. Depression was diagnosed on the basis of the respondents' answers to the questionnaire with no clinical diagnosis by a psychiatrist. Although the accuracy of the diagnosis may be questioned, the questionnaire included questions on a wide range of phenotypic traits, and respondents could not associate them with any diagnoses. Data from biobanks or mass genotyping services such as 23 and Me allowed them to markedly increase the sample size. For example, the study by Hyde et al. (131) included >450,000 individuals, and analysis of their questionnaire data allowed them to diagnose depression in about 120,000 participants (131). Samples of this size are an order of magnitude greater than those included in the PGC studies or CONVERGE Project, and help to minimize the problems caused by DD diagnostic errors. The authors managed to identify 17 SNP markers in 15 loci whose significance level was >5 × 10^−8^, which reflects the size of the sample analyzed. The DNA markers detected differ from those associated with DD in the PGC studies, although they both analyzed samples of European origin. Therefore, the problem of the reproducibility of results obtained in the GWASs remains to be solved.

A possible way to solve this problem is to conduct a meta-analysis of GWA studies. This analysis was carried out by Wray et al. (121). This meta-analysis identified 44 independent loci that were statistically significant (*P* < 5 × 10^−8^). Of these loci, 30 are new and 14 were significant in a prior study of MDD or depressive symptoms, and 6 shared loci with schizophrenia. Thus, the increase in sample sizes in the meta-analysis, on the one hand, allows the confirmation of the results obtained earlier with GWAS for the previously described loci associated with MDD. On the other hand, it increases the power of the study, by increasing the sample size, making it possible to identify new loci associated with the MDD.

Several methods were proposed for calculating of genetic risk score (GRS): simple count genetic risk score (SC-GRS), odds ratio weighted genetic risk score (OR-GRS), direct logistic regression genetic risk score (DL-GRS), polygenic genetic risk score (PG-GRS) and explained variance weighted genetic risk score (EV-GRS). Currently, the most widely used method is polygenic risk score (PGRS) (132). This approach has been used to obtain evidence of a genetic effect even when no single markers are significant, to establish a common genetic basis for related disorders, and to construct risk prediction models (133). Currently, alternative approaches to statistical analysis of GWASs data are proposed, where the analysis is not of individual DNA markers, but their combinations. Recently, several papers have been published using PGRS for the MDD and other psychiatric disorders (134–136). The possibility of using the PGRS to evaluate the cumulative contribution of several polymorphic variants of genes to the formation of endophenotypes of MDD was demonstrated. Whalley et al. (135), using the PGRS, divided the MDD into two subtypes, one of which is close to schizophrenia (135).

## Conclusions

Summing up the last quarter-century of investigation of the role of genetic factors in DD onset and progression, we note the polygenicity of inherited diseases with no pronounced effect of the principal gene. This has been made clear by recent studies undertaken in the CONVERGENCE and PGC studies and by analysis of candidate genes, which show that each of the genes implicated probably make a small contribution to DD progression. The important role of intergenic interactions has not been studied to date and will require new methods to analyze the data of association studies by including the contribution of combinations of two or more polymorphic DNA markers.

As noted above, DD typically has a high phenotypic heterogeneity, which may be manifested in differing severity of the main symptoms, and this heterogeneity limits the use of association studies. An approach to analyzing very large samples with minimally rigid assessment criteria for the DD phenotype (an extreme example is the variant identified by the 23andMe company associated with self-diagnostics of DD) is one possibility, but this must be supplemented by analysis of small and clinically highly homologous samples. This kind of analysis may not clear up the uncertainty about the genetics of DD on the whole, but it will make it possible to identify certain genetic variants underlying individual subphenotypes of DD.

A small but growing body of evidence suggests that mitochondrial dysfunction may play a role in the development of MDD. MDD was found to be associated with an increased production of mtROS, which could indicate a dysfunction of mitochondria (137, 138). Gardner et al. (139) found a decrease of mitochondrial ATP production rates and mitochondrial enzyme ratios in the muscle of patients with major depressive disorder and chronic physical conditions compared to controls (139). As well, decreased levels of an important part of electron transport chain, i.e., CoQ10, were found in the serum and peripheral blood mononuclear cells received from patients with MDD, which may also indicate a dysfunction of mitochondria (140).

Currently, however, there have been few studies on the role of genetic variants in mitochondrial DNA associated with MDD. A deletion in mtDNA in a child was associated with mitochondrial disease symptoms and with mild-moderate unipolar depression (141). Sequeira et al. (142) have analyzed post-mortem brain samples from human subjects and were failed to show associations of the mitochondrial haplogroups and major depression. However rare homoplasmic mutations with possible functional consequences were reveled in major depression cases, in the ATP synthase 8 (ATP8), ATP synthase 6 (ATP6), ND5 and cytochrome b (CYTB) genes, while another subject with depression demonstrated subthreshold heteroplasmy rate at a variant in the displacement loop (D-loop) part of mtDNA (142). Veronese et al. (143) found no significant associations between specific mitochondrial haplogroups and depressive symptoms either (143). Thus, changes in the functioning of mitochondria may be caused both by an abnormality of the mitochondrial DNA, and by variants of nuclear genes that encode mitochondrial proteins. The meta-analysis conducted by Huo et al. (141) identified *SCL25A37* as a novel MDD risk gene, and Zhang et al. (144) have showed that a haplotype T-C consisting of rs12457810 and rs12964485 in the 5'-upstream region of *NDUFV2* may be a protective factor for the development of MDD in Han Chinese (144, 145).

It is also important to supplement the association studies of inherited genome variants with analysis of dynamic modifications occurring during DD. There is much interest in the epigenetic changes that can modify a gene's functional status without changing its coding sequence. These epigenetic modifications can be caused by the action of different factors and can be stably inherited after disappearance of the factor causing the change. These epigenetic factors primarily involve DNA methylation and histone modification (methylation and acetylation). In recent years, several studies have analyzed changes in DNA methylation in DD. The first genome-wide analysis of methylation profiles in DD was by Sabuncian et al. (146), who assessed DNA methylation in *postmortem* frontal cortex material from DD patients and healthy people. In a number of regions, methylation differed reliably between healthy individuals and DD patients (146). A subsequent replicative investigation confirmed this modification of the methylation status in DD patients of the proline rich membrane anchor 1 gene, *PRIMA1*, which codes for the protein responsible for the assembly of acetylcholine esterase into tetramers and its “anchorage” in the neuron cellular membranes (147). This gene has not been mentioned in association with DD onset. Methylome changes were reported in peripheral blood of twins discordant with regard to DD occurrence. This study also reported on associative studies showing that, in some cases, the changes were related to genes linked to the development of the disorder (e.g., *ZBTB20, AGTPB1, TBC1D8*, and *CLSTN1*) (148). A number of studies have focused on associations between DD and histone methylation or acetylation (149, 150). Changes in lysine methylation of histone K27H3 were found *postmortem* in the *BDNF* promotor region in the prefrontal and frontal cortex of DD patients, and these changes correlated well with the expression level of *BDNF*. However, in this review, we do not analyze in detail the role of epigenetic factors in the development of the pathogenesis of DD.

Investigation of changes in the epigenome, transcriptome, and proteome in DD is probably limited by the nature of this disease and the need for brain tissue, which is possible only *postmortem*. Researchers must work with an extremely limited number of samples from patients, many of whom are on long-term treatment for both DD and somatic conditions. Therefore, to understand fully the entire process involved in DD onset and progression, studies of DD pathogenesis must be supplemented with experiments using different animal models of depression, which would permit an evaluation at different levels of the nervous system organization.

A threefold approach that combines gene-association studies with assessment of the epigenetic status of DD patients and analysis of the changes in animal models of depression, despite the limitations of such models (34), will enable researchers to identify the contributions of genetic, epigenetic, and environmental factors to different forms of DDs and to develop ways to reduce the risk of depression and to provide adequate treatment.

## Author contributions

All authors listed have made a substantial, direct and intellectual contribution to the work, and approved it for publication.

### Conflict of interest statement

The authors declare that the research was conducted in the absence of any commercial or financial relationships that could be construed as a potential conflict of interest.
